# Atypical fungal obstruction of the left ventricular assist device outflow cannula

**DOI:** 10.1186/1749-8090-8-S1-P36

**Published:** 2013-09-11

**Authors:** O Szarszoi, J Pirk, D Turek, Z Dorazilova, T Kotulak, H Riha, I Netuka, J Maly

**Affiliations:** 1 Department of Cardiac Surgery, Institute for Clinical and Experimental Medicine, Prague, Czech Republic; 2 Department of Cardiology, Institute for Clinical and Experimental Medicine, Prague, Czech Republic; 3 Department of Cardiac Anaesthesiology, Institute for Clinical and Experimental Medicine, Prague, Czech Republic

## Background

Even if better clinical outcomes have been achieved with the implantation of newer generation continuous-flow left ventricular assist devices (LVADs), infection complications are still a major risk for these patients in long-term follow-up.

We present a case of a 56-year-old male with dilated cardiomyopathy who was urgently implanted with left ventricular assist device HeartMate II. During the entire postoperative period, we observed repeated life-threatening septic complications. Clinical signs of infection disappeared after prolonged treatment with broad-spectrum antibiotics and patient was discharged to outpatient monitoring. During the entire duration of circulatory support, no LVAD suction events were detected, pump power consumption remained in the normal range and the patient was listed for heart transplantation. Patient was two month rehospitalized due to worsening of his status. Hemoglobinuria, an increase of inflammatory markers, and alteration in hepatic and renal function were detected. TEE imaging showed no obstructive formation in inflow or outflow cannulas; only velocities were decreased. The LV was severely dilated and the aortic valve was opening at each contraction. Patient was initially considered for systemic thrombolysis, but this urgent status was finally solved with explantation of the LVAD and subsequent heart transplantation. Massive obstruction with thrombus-like formation was found in the outflow cannula. Histopathologic examination and microbiologic culturing of the mass showed extensive fungal growth (Aspergillus species). During the postoperative period, patient was aggressively treated with antifungal therapy. He was discharged 8 weeks after the transplantation.

## Conclusion

We believe that clinical decision-making in patients with signs of ongoing sepsis and end-organ dysfunction together with any signs of LVAD malfunction should be very straightforward in terms of device exchange or explantation.

